# The impact of inhalation injury on fluid resuscitation in major burn patients: a 10-year multicenter retrospective study

**DOI:** 10.1186/s40001-024-01857-w

**Published:** 2024-05-12

**Authors:** Shuao Xiao, Zeping Pan, Hang Li, Yuheng Zhang, Tian Li, Hao Zhang, Jinbin Ning

**Affiliations:** 1 Department of Plastic and Burn Surgery, Second Affiliated Hospital of Air Force Medical University, 569 Xinsi Road, Baqiao District, Xi’an, 710038 China; 2 Department of Plastic and Burn Surgery, Joint Logistics Support Force of Chinese PLA, No. 927 Hospital Bao Yun Road, Puer, 665000 Yunnan China; 3 Department of Orthopedics, Western Theater Air Force Hospital of PLA, Chengdu, 610011 China; 4 School of Basic Medicine, Fourth Military Medical University, 169 Changle West Rd, Xi’an, 710032 China

**Keywords:** Inhalation injury, Fluid resuscitation, TMMU protocol, Major burn

## Abstract

**Background:**

It remains unclear whether additional fluid supplementation is necessary during the acute resuscitation period for patients with combined inhalational injury (INHI) under the guidance of the Third Military Medical University (TMMU) protocol.

**Methods:**

A 10-year multicenter, retrospective cohort study, involved patients with burns ≥ 50% total burn surface area (TBSA) was conducted. The effect of INHI, INHI severity, and tracheotomy on the fluid management in burn patients was assessed. Cumulative fluid administration, cumulative urine output, and cumulative fluid retention within 72 h were collected and systematically analyzed.

**Results:**

A total of 108 patients were included in the analysis, 85 with concomitant INHI and 23 with thermal burn alone. There was no significant difference in total fluid administration during the 72-h post-burn between the INHI and non-INHI groups. Although no difference in the urine output and fluid retention was shown in the first 24 h, the INHI group had a significantly lower cumulative urine output and a higher cumulative fluid retention in the 48-h and 72-h post-burn (all *p* < 0.05). In addition, patients with severe INHI exhibited a significantly elevated incidence of complications (Pneumonia, 47.0% vs. 11.8%, *p* = 0.012), (AKI, 23.5% vs. 2.9%, *p* = 0.037). For patients with combined INHI, neither the severity of INHI nor the presence of a tracheotomy had any significant influence on fluid management during the acute resuscitation period.

**Conclusions:**

Additional fluid administration may be unnecessary in major burn patients with INHI under the guidance of the TMMU protocol.

## Introduction

Inhalation injury (INHI) refers to damage to the respiratory tract caused by inhaling hot air, smoke, toxic gases, steam, combustible vapors, or particles in the mouth, nose, throat, and trachea [1]. Approximately 10–20% of burn patients also suffer from INHI, which has been identified as an independent predictor of mortality in most epidemiological surveys of burn injuries [2, 3]. Shock is the leading cause of death during the acute resuscitation period, with most deaths occurring within the first 72 h after injury [4]. Therefore, prompt and effective fluid resuscitation is of paramount importance in managing shock and improving patient outcomes. While poor administration of resuscitation fluid can lead to adverse clinical outcomes, excessive fluid volumes are associated with an increased risk of morbidity and life-threatening complications [5].

Despite the use of various fluid resuscitation methods, such as the Parkland, Evans, and Brook formulas, there is no international consensus on fluid resuscitation protocols in burn care. This has resulted in considerable variation in fluid management practices across different regions and burn center guidelines. In China, the Third Military Medical University (TMMU) formula has gained widespread acceptance for fluid resuscitation during the early phase of severe burn treatment [6]. Notably, the TMMU protocol differs from other fluid resuscitation protocols primarily in its early use of colloids [7]. However, limited research has been conducted on the effects of INHI on fluid resuscitation during the shock phase under the guidance of the TMMU protocol.

Debates have been ongoing for decades regarding whether burn patients with combined INHI should receive more fluid resuscitation than those with similar thermal injuries but no respiratory tract involvement. The inclusion criteria for %TBSA have varied widely in previous studies, and the lack of a unified criterion for %TBSA inclusion may be one reason for the differing conclusions reached. Furthermore, patients with smaller burn areas may have the ability to maintain fluid balance and normal tissue perfusion through compensatory mechanisms, potentially leading to higher tolerance for insufficient or excessive fluid replacement, which makes it difficult to assess the impact of INHI on fluid resuscitation needs. It is, therefore, imperative to investigate whether the presence of INHI results in additional fluid resuscitation requirements in burn patients with larger %TBSA. In addition, the severity of INHI and whether a tracheostomy was performed may also affect fluid resuscitation volume during the shock phase [8, 9].

Thus, we aimed to investigate the influence of INHI, INHI severity, and tracheotomy on the fluid management during acute resuscitation phase in burn patients with TBSA ≥ 50% guided by the TMMU protocol.

## Methods

### Study population

**Fig. 1:**
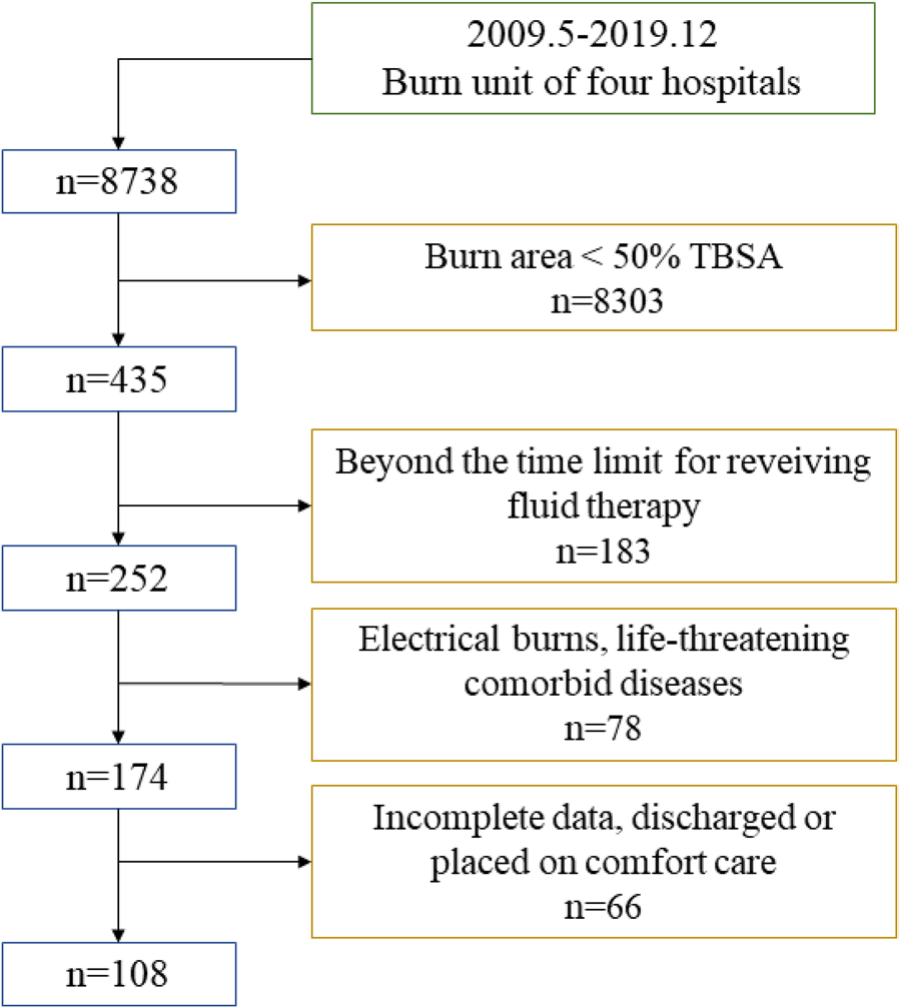
Flow chart of this study

We conducted a multicenter retrospective study, selecting four tertiary hospitals in the northwest of China: Second Affiliated Hospital of Air Force Medical University, Shaanxi Provincial People's Hospital, Xi'an Central Hospital, and Yan’an University First Affiliated Hospital. We enrolled patients who suffered from severe thermal injury between May 2009 and December 2019 and met the following inclusion criteria (Fig. 1): %TBSA burned of ≥ 50%; age ≥ 18 years; complete data; the time between thermal injury and fluid therapy was less than 4 h; and arrival at the BICU less than 6-h post-burn. Patients were excluded if they met any of the following criteria: electrical burns; pre-existing hepatic, respiratory, cardiac, neural or renal dysfunction, pre-existing coagulopathy, or those requiring compassionate care only; patients who died upon admission or refused any treatment.

Upon admission, patients were routinely given high-concentration oxygen therapy or high-flow nasal oxygen therapy. Mechanical ventilation was initiated under the following conditions: continued deterioration despite active treatment; disorders of consciousness; severe abnormal breathing patterns, such as a respiratory rate > 35–40 times/min or < 6–8 times/min, irregular rhythm, weak or absent spontaneous breathing; blood gas analysis indicating severe impairment of ventilation and oxygenation: PaO_2_ < 50mmHg; progressive increase in PaCO_2_, dynamic decrease in pH. Patients without INHI were assisted with endotracheal intubation for ventilation, while those with INHI underwent tracheostomy for mechanical ventilation [10]. The initial mode of mechanical ventilation for major burn patients was synchronized intermittent mandatory ventilation (SIMV). The level of inspired oxygen was adjusted to maintain the patient's pulse oxygen saturation at 90–95% and PaO_2_ at 60–80 mmHg or above. For early treatment of patients with extensive burns, broad-spectrum antibiotics should be administered to prevent infection, enteral nutrition support should be initiated as early as possible, and airway lavage should be performed to remove harmful substances in cases of INHI. Early surgical intervention included prompt debridement, removal of necrotic tissue, and early extensive skin grafting to close the wound and reduce infection and fluid loss [10].

### Data sources

**Fig. 2:**
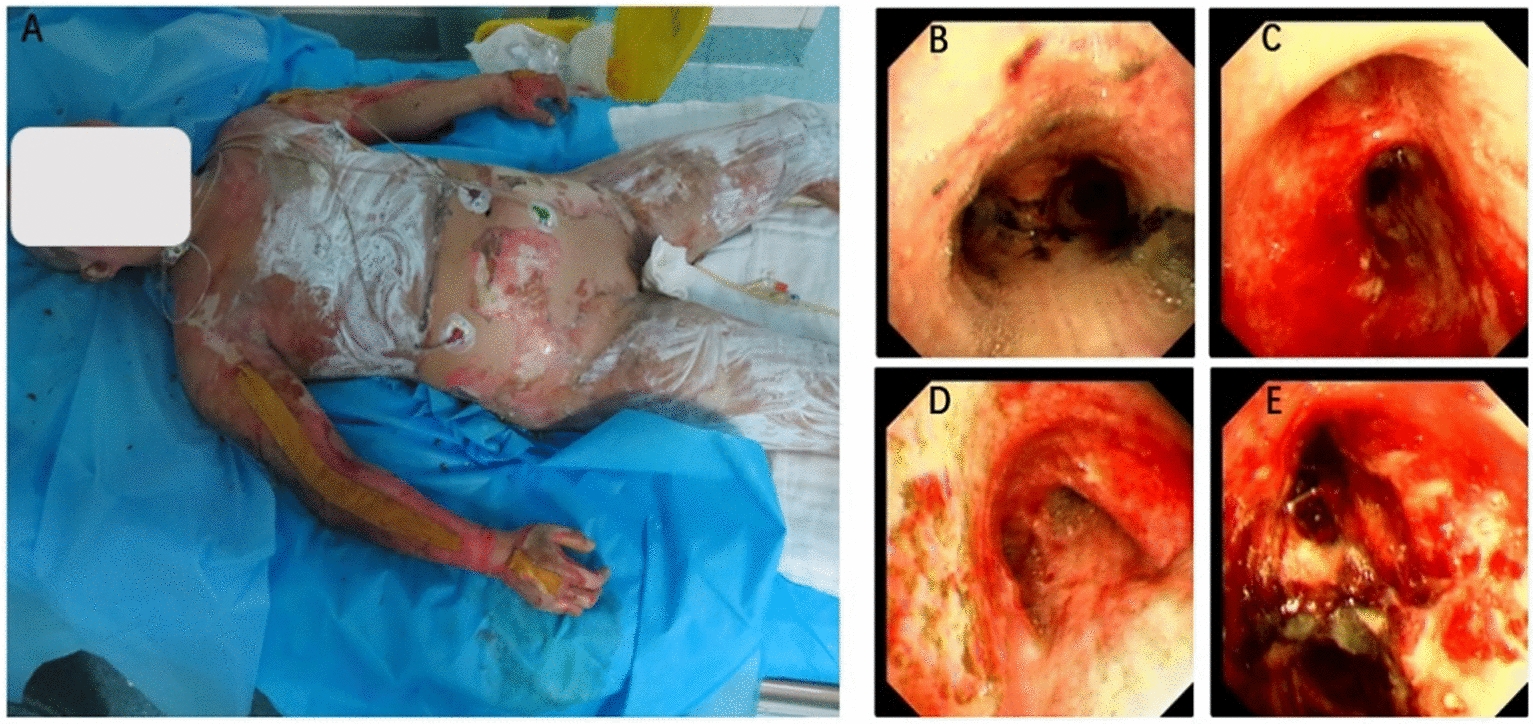
Representative photographs of patients with extensive burns and endoscopic images illustrating the degree of inhalation injury. **A** Patient with extensive burns; **B** Grade 1; **C** Grade 2; **D** Grade 3; **E** Grade 4

We retrospectively aggregated data from the electronic medical record system, categorizing it into demographics, clinical outcomes, and resuscitation-assessment parameters. Demographic information included gender, age, weight, %TBSA, %full-thickness burn, INHI grade, and mortality. Bronchoscopy was utilized to ascertain the diagnosis of INHI. Microscopic signs of airway congestion, edema, soot deposition, and mucosal sloughing were compelling evidence for the diagnosis of inhalation injury [11]. The degree of INHI was established from initial bronchoscopy findings, according to the Abbreviated Injury Score criteria [12]. The representative photographs of patients with extensive burns and endoscopic images illustrating the degree of inhalation injury were shown in Fig. 2. We also recorded the occurrence of tracheotomy within 24 h of admission. Clinical outcomes within 72 h of admission included the occurrence rates of pneumonia, acute respiratory distress syndrome (ARDS), acute kidney injury (AKI), and sepsis. Pneumonia was defined as a positive bronchoalveolar lavage (BAL) with > 100,000 colony forming units and clinical suspicion of pneumonia. The incidence of acute respiratory distress syndrome (ARDS) was defined according to the Berlin definition [13]. The incidence of acute kidney injury (AKI) was assessed according to AKIN definition [14]. Sepsis was diagnosed when hemodynamic instability coincided with documented evidence of infection from blood, urine, BAL, or wound samples. Resuscitation-assessment parameters included the cumulative fluid administration volume (total of all fluids administered, orally and intravenously), the cumulative urine output volume, and the cumulative fluid retention volume during 24-, 48-, and 72-h post-burn. To calculate the fluid retention, the formula described by Boer et al. was applied: Cumulative fluid retention = (cumulative fluid administration volume–cumulative urine output volume)/weight /%TBSA, where weight refers to the weight prior to hospitalization [9].

### TMMU fluid resuscitation

Patients in this study were given fluid resuscitation therapy based on the established TMMU protocol [6]. The initial fluid requirements were calculated as 1.5 ml/kg/% TBSA + 2000 ml for the first 24 h and 0.75 ml/kg/% TBSA + 2000 ml for the second 24-h post-burn. Lactated Ringer’s solution was used as a crystalloid, and the frozen plasma or 5% albumin solution was given as a colloid. The ratio of colloid to crystal volumes was expected to be 1:2. An additional 2000 ml of 5% glucose solution was administered daily to meet the patient's physical demand for water. One-half of the calculated crystalloid solution and colloid should be administered within the first 8h post-burn, with the remainder given homogeneously over the next 16 h. Subsequently, fluid resuscitation was guided based on clinical parameters. The TMMU formula only provides an estimation of the required fluid volume for burn patients. The fluid resuscitation should be individualized based on the situation of patient, such as age, gender, burn depth, patient response, and overall condition. Specifically, the rate of fluid resuscitation should be adjusted according to a comprehensive assessment of the patient and monitoring of urine output, vital signs, mental status, and tissue perfusion [6].

### Statistical analysis

Continuous variables are reported as means and standard deviations or medians and interquartile ranges, while dichotomous and categorical variables are presented as counts and relative percentages in each group. The student’s *t* test was used to analyze differences between groups with normally distributed data, while the Mann–Whitney *U* test was performed to analyze differences between groups with non-normally distributed data. Pearson’s Chi-squared test or Fisher’s exact test were also used to examine differences between categorical data, frequencies or rates. To adjust for intergroup difference (INHI vs. non-INHI; high-grade INHI vs. low-grade INHI), a case–control matching analysis was conducted using a one-to-two matching without replacement approach. Further analyses between groups were performed before and after matching. For all analyses, *p* < 0.05 was considered to suggest statistical significance. All procedures were conducted using SPSS (version 25.0; IBM, USA) and R software (version 3.6.1; R Foundation for Statistical Computing, Vienna, Austria).

## Results

### Patient characteristics and outcomes when grouped by INHI

A total of 108 patients were enrolled in the study, with 85 patients having concomitant INHI and 23 patients having a thermal burn alone. The cohort was predominantly male (82.4%) with a mean age of 40.6 ± 11.6, a mean weight of 67.3 ± 10.4, and a median %TBSA of 70 (55–84.5). Of these 85 patients with INHI, 24 patients had grade 1 injury, 38 patients had grade 2 injury, 20 patients had grade 3 injury, and 3 patients had grade 4 injury. To evaluate the effect of INHI on fluid resuscitation in severe burn patients, we first classified patients into INHI and non-INHI groups. Patient characteristics by group are reported in Table 1. There were no significant differences in gender, age, weight, or mortality between the two groups. However, the %TBSA in the INHI group was significantly larger than that in the non-INHI group (71.2% vs. 61.3%, *p* = 0.008). In addition, %full-thickness burn was also found larger in the INHI group, but without reaching statistical significance (37.8% vs. 27.5%, *p* = 0.080). To adjust for intergroup differences, we conducted a case–control matching analysis performing one-to-two matching (non-INHI group to INHI group) without replacement in terms of gender, age, weight, %TBSA and %full-thickness burn. After matching, 20 patients of the non-INHI group and 40 patients of the INHI group were identified, and no significant difference in demographic variables was demonstrated (Table 1). Regarding morbidity, the incidence of pneumonia was significantly higher in the INHI group before and after matching (24.7% vs. 4.3%, *p* = 0.039; 27.5% vs. 5.0%, *p* = 0.047), whereas no difference was found in the incidence of ARDS, AKI and sepsis.

**Table 1:** Patient demographics and outcomes grouped by INHI

Demographics	Before match			After match		
	INHI	Non-INHI	*p* value	INHI	Non-INHI	*p* value
No. of patients	85	23		40	20	
Sex (male), n (%)	72 (84.7)	17 (73.9)	0.082	33 (82.5)	16 (80.0)	1.000
Age (years)	41.0 ± 11.4	39.1 ± 12.5	0.490	40.8 ± 11.1	38.6 ± 12.6	0.502
Weight (kg)	67.5 ± 10.5	66.3 ± 10.2	0.620	68.7 ± 11.3	66.8 ± 9.6	0.528
TBSA (%)	71.2 (55–85)	61.3 (50–65)	0.008	62.3 (51.5–70)	62.5 (53.8–66.3)	0.960
Full-thickness burn (%)	37.8 (18–60)	27.5 (15–34)	0.080	29.2 (13.8–42.8)	28.4 (15–35.5)	0.898
Mortality, n (%)	22 (25.9)	2 (8.7)	0.095	5 (12.5)	2 (10.0)	1.000
Etiology of burn injury, n (%)						
Flame	85 (100)	3 (13.0)		40 (100)	2 (10.0)	
Hot water	0 (0)	20 (87.0)		0 (0)	18 (90.0)	
Morbidity						
Pneumonia, n (%)	21 (24.7)	1 (4.3)	0.039	11 (27.5)	1 (5.0)	0.047
ARDS, n (%)	12 (14.1)	1 (4.3)	0.292	5 (12.5)	1 (5.0)	0.653
AKI, n (%)	6 (7.1)	1 (4.3)	1.000	4 (10.0)	1 (5.0)	0.656
Sepsis, n (%)	3 (3.5)	3 (13.0)	0.110	2 (5.0)	3 (15.0)	0.322

Data are reported as means ± standard deviations or medians and interquartile ranges for continuous variables, number (%) for categorical variables. Age, weight, %TBSA and %full-thickness burn were analyzed using student’s *t* test or Mann–Whitney *U* test. Sex, mortality and morbidity were analyzed using Fisher’s exact test. The non-INHI group and INHI groups were well-matched in terms of gender, age, weight, TBSA, and extent of full-thickness burns at a ratio of 1:2 without replacement

*INHI* inhalation injury, *TBSA* total body surface area, *ARDS* Acute Respiratory Distress Syndrome, *AKI*, acute kidney injury

### Patient resuscitation-assessment parameters when grouped by INHI

There was no significant difference between the two groups in total fluid administration during the 72-h post-burn period. Although no difference in the urine output and fluid retention was observed in the first 24 h, the INHI group had a significantly lower cumulative urine output and a higher cumulative fluid retention in the 48-h and 72-h post-burn after matching, when compared to the non-INHI group (all *p* < 0.05) (Table 2).

**Table 2:** Patient resuscitation-assessment parameters grouped by INHI

	Before match			After match		
	INHI	Non-INHI	*p* Value	INHI	Non-INHI	*p* value
24 h						
Fluid requirement (ml/kg/%TBSA)	2.2 ± 0.5	2.2 ± 0.5	0.994	2.3 ± 0.5	2.2 ± 0.5	0.370
Urine output (ml/kg/h)	0.8 ± 0.3	0.8 ± 0.3	0.399	0.7 ± 0.3	0.8 ± 0.3	0.194
Fluid retention (ml/kg/%TBSA)	1.9 ± 0.5	1.8 ± 0.6	0.631	2.0 ± 0.5	1.9 ± 0.5	0.352
48 h						
Fluid requirement (ml/kg/%TBSA)	3.6 ± 0.8	3.6 ± 0.8	0.916	3.8 ± 0.8	3.7 ± 0.7	0.264
Urine output (ml/kg/h)	1.3 ± 0.4	1.5 ± 0.6	0.012	1.2 ± 0.4	1.6 ± 0.6	0.003
Fluid retention (ml/kg/%TBSA)	2.7 ± 0.8	2.4 ± 0.8	0.062	2.8 ± 0.8	2.3 ± 0.7	0.032
72 h						
Fluid requirement (ml/kg/%TBSA)	4.8 ± 1.1	4.8 ± 1.0	0.876	5.0 ± 1.0	4.8 ± 1.0	0.165
Urine output (ml/kg/h)	1.6 ± 0.4	1.9 ± 0.7	0.011	1.6 ± 0.4	2.0 ± 0.7	0.011
Fluid retention (ml/kg/%TBSA)	3.1 ± 1.0	2.5 ± 1.1	0.009	3.2 ± 1.0	2.5 ± 1.1	0.020

Data are reported as means ± standard deviations. Fluid requirement, urine output and fluid retention were analyzed using student’s *t* test. The non-INHI group and INHI groups were well-matched in terms of gender, age, weight, TBSA, extent of full-thickness burns

*INHI* inhalation injury, *TBSA* total body surface area

### Patient characteristics and outcomes when grouped by INHI severity

To assess the impact of INHI severity on fluid administration requirements, we further categorized patients with concomitant INHI (*n* = 85) into low-grade (grades 1 and 2) (*n* = 62) and high-grade (grades 3 and 4) (*n* = 23) groups. As shown in Table 3, the high-grade INHI group had a significantly larger burn surface area than those in the low-grade INHI group (%TBSA, 78.9% vs. 68.3%, *p* = 0.007), (%full-thickness burn, 53.5% vs. 32%, *p* = 0.001). To control for intergroup bias, we also performed a case–control matching analysis at a ratio of 1:2 based on clinical parameters. A total of 17 patients belonging to the high-grade INHI group and 34 patients belonging to the low-grade INHI group were identified. After matching, no significant difference in demographic variables was reported (Table 3). The high-grade INHI group showed a significantly elevated incidence of complications (Pneumonia, 47.0% vs. 11.8%, *p* = 0.012), (ARDS, 23.5% vs. 5.9%, *p* = 0.087), (AKI, 23.5% vs. 2.9%, *p* = 0.037).

**Table 3:** Patient demographics and outcomes grouped by INHI severity

Demographics	Before match			After match		
	High-grade	Low-grade	*p* value	High-grade	Low-grade	*p* value
No. of patients	23	62		17	34	
Sex (male), n (%)	21 (91.3)	51 (82.3)	0.719	16 (94.1)	27 (79.4)	0.242
Age (years)	39.3 ± 7.1	41.6 ± 12.7	0.400	39.4 ± 7.0	40.9 ± 10.6	0.593
Weight (kg)	68.2 ± 12.5	67.3 ± 9.8	0.716	68.9 ± 13.3	66.9 ± 9.0	0.541
TBSA (%)	78.9 (70–90)	68.3 (54.3–80)	0.007	73.4 (65–85)	72.4 (60–82.3)	0.817
Full-thickness burn (%)	53.5 (35–71.5)	32 (15–49)	0.001	42.9 (25–60)	42.3 (20.8–60)	0.927
Mortality, n (%)	8 (34.8)	14 (22.6)	0.275	3 (17.6)	8 (23.5)	0.731
Morbidity						
Pneumonia, n (%)	9 (39.1)	12 (19.4)	0.111	8 (47.0)	4 (11.8)	0.012
ARDS, n (%)	5 (21.7)	7 (11.3)	0.293	4 (23.5)	2 (5.9)	0.087
AKI, n (%)	4 (17.4)	2 (3.2)	0.043	4 (23.5)	1 (2.9)	0.037
Sepsis, n (%)	2 (8.7)	1 (1.6)	0.177	1 (5.9)	0	0.333

Data are reported as means ± standard deviations or medians and interquartile ranges for continuous variables, number (%) for categorical variables. Age, weight, %TBSA and %full-thickness burn were analyzed using student’s *t* test or Mann–Whitney *U* test. Sex, mortality and morbidity were analyzed using Fisher’s exact test. The high-grade INHI group and low-grade INHI groups were well-matched in terms of gender, age, weight, TBSA, extent of full-thickness burns at a ratio of 1:2 without replacement

*INHI* inhalation injury, *TBSA* total body surface area, *ARDS* Acute Respiratory Distress Syndrome, *AKI*, acute kidney injury

### Patient resuscitation-assessment parameters when grouped by INHI severity

As shown in Table 4, there was no difference in fluid administration, urine output, or fluid retention during the 72-h post-burn between patients with low-grade and high-grade INHI, before and after matching (all *p* > 0.05).

**Table 4:** Patient resuscitation-assessment parameters grouped by INHI severity

	Before match			After match		
	High-grade	Low-grade	*p* value	High-grade	Low-grade	*p* Value
24 h						
Fluid requirement (ml/kg/%TBSA)	2.2 ± 0.6	2.1 ± 0.5	0.667	2.2 ± 0.6	2.2 ± 0.6	0.974
Urine output (ml/kg/h)	0.8 ± 0.3	0.8 ± 0.4	0.706	0.9 ± 0.2	0.7 ± 0.4	0.192
Fluid retention (ml/kg)	1.9 ± 0.6	1.9 ± 0.5	0.562	1.9 ± 0.6	1.9 ± 0.6	0.755
48 h						
Fluid requirement (ml/kg/TBSA)	3.7 ± 0.9	3.6 ± 0.8	0.578	3.8 ± 1.0	3.8 ± 0.8	0.962
Urine output (ml/kg/h)	1.3 ± 0.3	1.3 ± 0.4	0.390	1.4 ± 0.3	1.3 ± 0.5	0.516
Fluid retention (ml/kg)	2.9 ± 0.9	2.7 ± 0.8	0.330	2.8 ± 1.0	2.9 ± 0.9	0.844
72 h						
Fluid requirement (ml/kg/TBSA)	5.0 ± 1.2	4.8 ± 1.0	0.400	5.1 ± 1.3	5.0 ± 1.0	0.795
Urine output (ml/kg/h)	1.7 ± 0.3	1.6 ± 0.4	0.198	1.7 ± 0.4	1.7 ± 0.5	0.805
Fluid retention (ml/kg)	3.4 ± 1.1	3.0 ± 0.9	0.135	3.4 ± 1.2	3.2 ± 1.1	0.717

Data are reported as means ± standard deviations. Fluid requirement, urine output and fluid retention were analyzed using student’s *t* test. The high-grade INHI group and low-grade INHI groups were well-matched in terms of gender, age, weight, TBSA, extent of full-thickness burns at a ratio of 1:2 without replacement

*INHI* inhalation injury, *TBSA* total body surface area

### INHI patient characteristics and outcomes when grouped by tracheotomy

Patients with combined INHI often require prophylactic tracheostomy to prevent airway obstruction. We sought to determine whether there was a difference in fluid management between the tracheostomy and non-tracheostomy groups, and 85 patients were subsequently divided into two groups: tracheostomy and non-tracheostomy. As presented in Table 5, the tracheotomy group showed a larger %full-thickness burn than that in the non-tracheotomy group, but without reaching statistical difference (median 41 vs. 31.1, *p* = 0.104). The two groups did not differ in other variables and showed no difference in overall morbidity and mortality.

**Table 5:** INHI patient demographics and outcomes grouped by tracheotomy

Demographics	All	Tracheotomy	Non-tracheotomy	*p* value
No. of patients	85	58	27	
Sex (male), n (%)	68 (80)	50 (86.2)	22 (81.5)	0.243
Age (years)	41.0 ± 11.4	41.5 ± 10.7	39.9 ± 13.0	0.562
Weight (kg)	67.5 ± 10.5	67.6 ± 10.9	67.4 ± 9.8	0.960
TBSA (%)	71.2 (55–85)	72.4 (55–89)	68.7 (55–80)	0.329
Full-thickness burn (%)	37.8 (18–60)	41 (19.3–60)	31.1 (15–43.5)	0.104
Mortality, n (%)	22 (25.9)	15 (25.9)	7 (25.9)	1.000
Morbidity				
Pneumonia, n (%)	21 (24.7)	16 (27.6)	5 (18.5)	0.666
ARDS, n (%)	12 (14.1)	7 (12.1)	5 (18.5)	0.694
AKI, n (%)	6 (7.1)	5 (8.6)	1 (3.7)	0.851
Sepsis, n (%)	3 (3.5)	3 (5.2)	0 (0.0)	0.644

Data are reported as means ± standard deviations or medians and interquartile ranges for continuous variables, number (%) for categorical variables. Age, weight, %TBSA and %full-thickness burn were analyzed using student’s *t* test or Mann–Whitney *U* test. Sex, mortality and morbidity were analyzed using Fisher’s exact test

*INHI* inhalation injury, *TBSA* total body surface area, *ARDS* Acute Respiratory Distress Syndrome, *AKI* acute kidney injury

### INHI patient resuscitation-assessment parameters when grouped by tracheotomy

There was no significant difference in cumulative fluid administration, urine output, or fluid retention between the two groups during the 72-h post-burn, between and after matching (all *p* > 0.05) (Table 6).

**Table 6:** INHI patient resuscitation-assessment parameters grouped by tracheotomy

	All	Tracheotomy	Non-tracheotomy	*p* value
24 h				
Fluid requirement (ml/kg/%TBSA)	2.2 ± 0.5	2.2 ± 0.6	2.1 ± 0.5	0.789
Urine output (ml/kg/h)	0.8 ± 0.3	0.8 ± 0.3	0.8 ± 0.4	0.508
Fluid retention (ml/kg/%TBSA)	1.9 ± 0.5	1.9 ± 0.6	1.8 ± 0.5	0.534
48 h				
Fluid requirement (ml/kg/TBSA)	3.6 ± 0.8	3.7 ± 0.9	3.6 ± 0.8	0.708
Urine output (ml/kg/h)	1.3 ± 0.4	1.3 ± 0.3	1.4 ± 0.6	0.705
Fluid retention (ml/kg/%TBSA)	2.6 ± 0.8	2.7 ± 0.8	2.5 ± 0.8	0.253
72 h				
Fluid requirement (ml/kg/TBSA)	4.8 ± 1.1	4.9 ± 1.1	4.8 ± 1.0	0.580
Urine output (ml/kg/h)	1.7 ± 0.5	1.7 ± 0.3	1.7 ± 0.6	0.761
Fluid retention (ml/kg/%TBSA)	3.0 ± 1.1	3.1 ± 1.0	2.8 ± 1.1	0.091

Data are reported as means ± standard deviations. Fluid requirement, urine output and fluid retention were analyzed using student’s *t* test

*INHI* inhalation injury, *TBSA* total body surface area

Discussion

### Main finding and interpretation

Severe burns are among the most critical injuries, and appropriate fluid resuscitation is critical in the early post-injury period. Under-resuscitation can lead to organ hypoperfusion, shock, and other hazards, but over-resuscitation can lead to the risk of serious complications, such as pneumonia, sepsis, and cardiopulmonary dysfunction [15]. Thus, investigating whether INHI increases the need for additional fluid resuscitation is of great significance for the management of major burn patients during the acute resuscitation phase. In this multicenter retrospective study, a total of 108 patients with %TBSA ≥ 50% were included in the analysis. We did not observe a significant increase in fluid resuscitation requirements in major burn patients with combined INHI. However, INHI may have an impact on fluid balance in major burn patients, as evidenced by lower cumulative urine output and higher cumulative fluid retention in the 48-h and 72-h post-burn in the INHI group. In addition, the severity of INHI and tracheotomy did not appear to have a significant impact on fluid management in patients with combined INHI.

Upon reviewing previous literature, we found that the inclusion criterion for %TBSA in related studies varied greatly, ranging from 0 to 30% [8, 16–20]. Some studies even lacked clear inclusion criterion for %TBSA [12]. This may be one of the reasons for the different conclusions drawn by these studies. To better assess the impact of INHI on fluid resuscitation requirements, we set a relatively high inclusion criterion of 50% TBSA. The reasons were as follows: First, in our clinical practice, we have noticed that patients with minor burns are able to maintain fluid balance and normal tissue perfusion through compensatory mechanisms, which can confer greater tolerance to suboptimal fluid replacement. However, patients with %TBSA ≥ 50% are faced with a more severe state of shock, and fluid resuscitation becomes a critical and essential treatment measure. Second, for patients with %TBSA ≥ 50%, fluid resuscitation requirements for shock management need to be more precise and carefully regulated to avoid adverse outcomes resulting from under- or over-resuscitation. Investigating whether INHI increases fluid resuscitation needs is particularly relevant and clinically informative in the context of burn injuries of this severity, as it highlights the impact of INHI on fluid resuscitation requirements. Third, another major complication of excessive fluid resuscitation is compartment syndrome of the limbs or abdomen. Excessive fluid resuscitation in severe burn patients can cause massive edema in burned and unburned tissues, increasing the risk of abdominal hypertension and subsequent abdominal compartment syndrome. Markell et al. found that patients with %TBSA ≥ 50% combined with INHI are more likely to develop abdominal compartment syndrome [5]. Thus, in this study, we established a 50% TBSA inclusion criterion to ensure that we were capturing patients in the most critical and relevant cases for fluid resuscitation.

The demographic data showed that patients with combined INHI had significantly larger and deeper burns, consistent with previous literature reports [16, 17, 20]. As burns increase in size and depth, patients typically require more fluid, but the increase in fluid requirements is not entirely linear, and there is great individual variability [21, 22]. To control for differences in burn area between groups, we used a case–control matching approach to assess the impact of INHI on fluid requirements in similar burn areas and depths. We found that patients in the combined INHI group did not require additional fluid resuscitation, even when subjects were divided into low-grade and high-grade groups. In addition, we observed that patients subjected to mechanical ventilation via tracheostomy exhibited a notable trend towards increased fluid retention, reaching near statistical significance by the third-day post-intervention. This phenomenon can be elucidated through several physiological mechanisms. First, the elevation in mean intrathoracic pressure associated with mechanical ventilation reduced venous return, leading to an increase in central venous pressure. Such changes imposed a greater afterload on the right ventricle, subsequently diminishing cardiac output and augmenting sympathetic nervous system activity [23]. Moreover, the application of positive pressure for lung expansion triggered an extensive endocrine response, characterized by elevated plasma levels of norepinephrine, increased renin activity, and a rise in atrial natriuretic peptide. These hormonal shifts contributed to heightened sympathetic tone, promoting renal retention of fluids [24]. Boer et al. reported that the increased fluid retention, traditionally linked with INHI, results from the effects of ventilation rather than from INHI itself [9].

Although there was no significant difference in fluid demand, we found that patients in the INHI group had significantly lower urine output and higher fluid retention. This indicates that although both groups of patients received the same amount of fluid resuscitation, the volume of fluid circulating in the INHI patients was significantly reduced. The physiological and pathological processes of INHI contributed to this finding. INHI is caused by direct local thermal injury and exposure to numerous toxic chemicals that can directly cause mucous shedding, bronchial edema, airway obstruction, hypoxia, and severe inflammatory reactions in the respiratory tract and even throughout the body [25]. These processes can lead to lung infections and increased blood vessel permeability, which worsens tissue edema and reduces circulating volume [2]. This is further supported by subsequent analysis of complications. INHI is associated with higher levels of local and systemic inflammation, and fluid overload, rather than INHI, may mediate the development of acute kidney injury in major burn patients [18, 26]. Therefore, we believe that additional fluid resuscitation may not be required for major burn patients with combined INHI. To avoid over-resuscitation, the appropriate use of high doses of vitamin C and hypertonic saline solution may play a more active role [27–29].

In the late twentieth century, it was believed that INHI had escalated fluid resuscitation needs of burn victims. However, in recent years, medical concepts and technologies have been advancing, and this view has been increasingly challenged. Endorff and Gamelli found no difference in the initial 24-h fluid requirements between 25 patients with grades 0 and 1 injury (6.6 ± 0.7ml/kg/%TBSA) and 35 patients with grades 2, 3, or 4 injury (6.7 ± 0.4ml/kg/%TBSA) [12]. Similarly, in a prospective observational study conducted by Albright et al., no significant association was observed between inhalation severity grades ranging from 0 to 4 and 24-h or 72-h fluid requirements, even when comparing patients with low-grade INHI (grades 1 and 2, *n* = 30) to those with high-grade INHI (grades 3 and 4, *n* = 21) [18]. Spano et al. also found no correlation between INHI severity grades and 24-h or 48-h fluid demand. Even when the INHI subjects were divided into low-grade (*n* = 78) and high-grade (*n* = 20) groups, no significant difference was discovered between low-grade (6.4 ml/kg/%TBSA) and high-grade (6.6 ml/kg/%TBSA) INHI in terms of the initial 24-h fluids [19]. Our findings are in line with those of prior studies. Tracheotomy was previously thought to increase fluid resuscitation requirements in patients, but we did not observe this phenomenon in major burn patients with combined INHI. This may be due to the fact that major burn patients require a large amount of fluid resuscitation, masking the small increase in fluid requirements caused by tracheotomy.

The fluid resuscitation strategy for managing burn shock is a significant milestone in burn care. During the 1960s and 1970s, multiple protocols and formulas were developed, including the Evans, Brooke, and Parkland formula, to provide guidelines for fluid resuscitation of burns. The TMMU formula, derived from the Evans formula, is widely used in China and is considered to be more appropriate for the Chinese population. This study showed that patients with TBSA ≥ 50% required an average of 2.4 ± 0.9 ml/kg/%TBSA of fluid within the first 24 h, which is significantly lower than what Spano et al. reported using the Parkland formula (6.1 ml/kg/%TBSA). This disparity can be attributed to the earlier emphasis of the TMMU formulation on colloidal applications, which are better able to maintain vessel volume and reduce fluid requirements. Tan et al. reported similar findings, where patients with TBSA ≥ 40% who received fluid resuscitation using the TMMU formula required an average of 2.35 ± 0.59 ml/kg/%TBSA of fluid during the first 24 h [22]. After excluding the possibility of excessive resuscitation based on urine output, our findings indicate that the TMMU fluid regimen underestimated the fluid requirements of patients with extensive burns. Notably, instances of fluid administration exceeding the calculated amount based on the formula also occurred in other studies using TMMU-based resuscitation protocols [22, 30]and crystalloid-based resuscitation formulas [31, 32].

### Limitations

There are several limitations to this study. First, despite being a multicenter retrospective study, our stringent inclusion criteria resulted in only 108 patients being included, potentially leading to a false negative result. Second, we did not evaluate lung function, pulmonary inflammation, or systemic inflammation levels, which could have provided additional insight into the pathophysiological mechanisms underlying our observations. Third, the estimation of fluid retention may not be accurate, as we did not subtract the daily explicit water loss, and the complexity of care for major burn patients may have influenced the calculation of fluid retention. Finally, Outcome differences may be due to the differing aetiologies of cause of burn, and secondary to differences in hemodynamics during resuscitation, for which we currently lack the corresponding data.

### Conclusion

Compared to non-INHI patients, patients with concomitant INHI may not require additional fluid resuscitation. In addition, the tracheostomy may not significantly affect fluid management in major burn patients with combined INHI. Further studies are necessary to explore fluid management in burn patients with INHI and to prevent potential complications while improving overall treatment outcomes.

## Data Availability

The data sets used and analyzed in this study are on https://www.jianguoyun.com/p/DdI-JZoQuaiFChjVlrEFIAA

## Abbreviations

INHI: Inhalation injury
TMMU: Third Military Medical University
TBSA: Total burn surface area
ARDS: Acute respiratory distress syndrome
AKI: Acute kidney injury
